# A Quantifiable 3D Closed-Loop Framework for Controlled Substance Safety in a HIMSS Electronic Medical Record Adoption Model (EMRAM) Stage 7 Hospital: Pre-Post Quality Improvement Study

**DOI:** 10.2196/98247

**Published:** 2026-09-25

**Authors:** Nasha Sun, Jihua Su, Qiyi Yu, Yong Xu, Mali Zhuo

**Affiliations:** 1 Ningbo Hospital of Integrated Traditional Chinese and Western Medicine Yinzhou District Second Hospital Ningbo, Zhejiang China

**Keywords:** controlled substances, medication safety, closed-loop management, digital health implementation, barcode administration, batch traceability, hospital information systems

## Abstract

**Background:**

Controlled substances require strict life cycle management, but manual workflows can lead to documentation errors, traceability gaps, and delays in empty-ampoule recovery.

**Objective:**

This study aimed to construct a quantifiable 3D closed-loop framework integrating technology, process redesign, and organizational accountability and to examine whether implementation was temporally associated with changes in controlled substance process indicators.

**Methods:**

A single-center pre-post quality improvement study was conducted in a Healthcare Information and Management Systems Society (HIMSS) Electronic Medical Record Adoption Model (EMRAM) stage 7 hospital. The preintervention period was September 2024 to November 2024, and the postintervention period was December 2024 to February 2025. The intervention combined unique drug identifiers, personal digital assistant (PDA)–based bedside verification, smart medication cabinets, predispensing prescription review rules, batch-number traceability, daily reconciliation, tiered alerts, staff training, and anomaly-handling standard operating procedures (SOPs). Outcomes were prescription dispensing record compliance, batch-number management noncompliance, failure to complete empty-ampoule recovery and electronic documentation within 24 hours, and implementation fidelity. Proportions were reported with Wilson 95% CIs. Between-period absolute differences and 95% CIs were calculated using the Wald normal approximation for independent proportions. Comparisons used the chi-square test or Fisher exact test, as appropriate.

**Results:**

The analysis included 3264 prescription dispensing records before implementation and 3311 prescription dispensing records after implementation. Prescription dispensing record compliance increased from 94.82% (3095/3264) to 98.7% (3268/3311). Batch-number management noncompliance decreased from 2.4% (24/1000) to 0.6% (6/1000). The rate of failure to complete empty-ampoule recovery and electronic documentation within 24 hours decreased from 5.61% (68/1213) to 0.78% (10/1274). Implementation fidelity indicators generated from operational logs were high across system stability, scanning execution, and anomaly closure.

**Conclusions:**

Implementation of the 3D closed-loop framework was temporally associated with improvements in controlled substance process indicators in a digitally mature hospital. The implementation parameters may serve as a reference for local feasibility testing in other wards or institutions, but transferability remains to be evaluated. Multicenter studies are still needed to confirm sustainability and transferability.

## Introduction

### Background

Before digitization, controlled substance management relied on paper logs, manual handoffs, and disconnected checks across the prescribing, dispensing, administration, and recovery stages. Global efforts to reduce medication-related harm have emphasized system-level redesign, and prior work has also documented persistent risks of controlled drug diversion and the need for broader patient safety safeguards [1-3]. These workflows are vulnerable to recording errors, weak end-to-end traceability, fragmented coordination, and delayed empty-ampoule recovery, highlighting the need for a fully digital closed-loop workflow [4].

### Prior Work

Although barcode technology has been applied to pharmaceutical management [5], existing research still has 3 important gaps. First, many implementations have focused on a single node, meaning 1 isolated point in the medication-use workflow, such as prescribing, dispensing, administration, or return, rather than the entire life cycle. Second, few studies have reported reproducible implementation parameters, which limits replication by other institutions. Third, empirical evidence remains limited regarding which components within a bundled closed-loop framework may plausibly contribute to observed improvements in controlled substance process indicators.

### Study Objective

The management of anesthetic drugs in health care institutions can be viewed as a sociotechnical system [6]. In a hospital that had achieved Healthcare Information and Management Systems Society (HIMSS) Electronic Medical Record Adoption Model (EMRAM) stage 7, we combined sociotechnical systems theory, the Systems Engineering Initiative for Patient Safety model, and closed-loop management theory [7-11] to build a quantifiable 3D framework spanning technology, process, and organization. We aimed to examine whether implementation of this bundled framework was temporally associated with changes in prescription dispensing record compliance, batch-number management, and failure of 24-hour empty-ampoule recovery and electronic documentation.

## Methods

### Study Design

This was a single-center, uncontrolled pre-post quality improvement study. The preintervention period was September 1, 2024, to November 30, 2024, during which anesthetic drugs were managed through the traditional manual workflow. The postintervention period was December 1, 2024, to February 28, 2025, after implementation of the barcode-enabled closed-loop management framework. The same institution, medication-use process, and target records were evaluated before and after implementation; therefore, the design describes temporal associations but cannot establish causality, as shown in Figure 1. Reporting was guided by SQUIRE 2.0 (Standards for Quality Improvement Reporting Excellence) for quality improvement studies and iCHECK-DH (Guidelines and Checklist for the Reporting on Digital Health Implementations) for digital health implementations [12,13].

**Figure 1:**
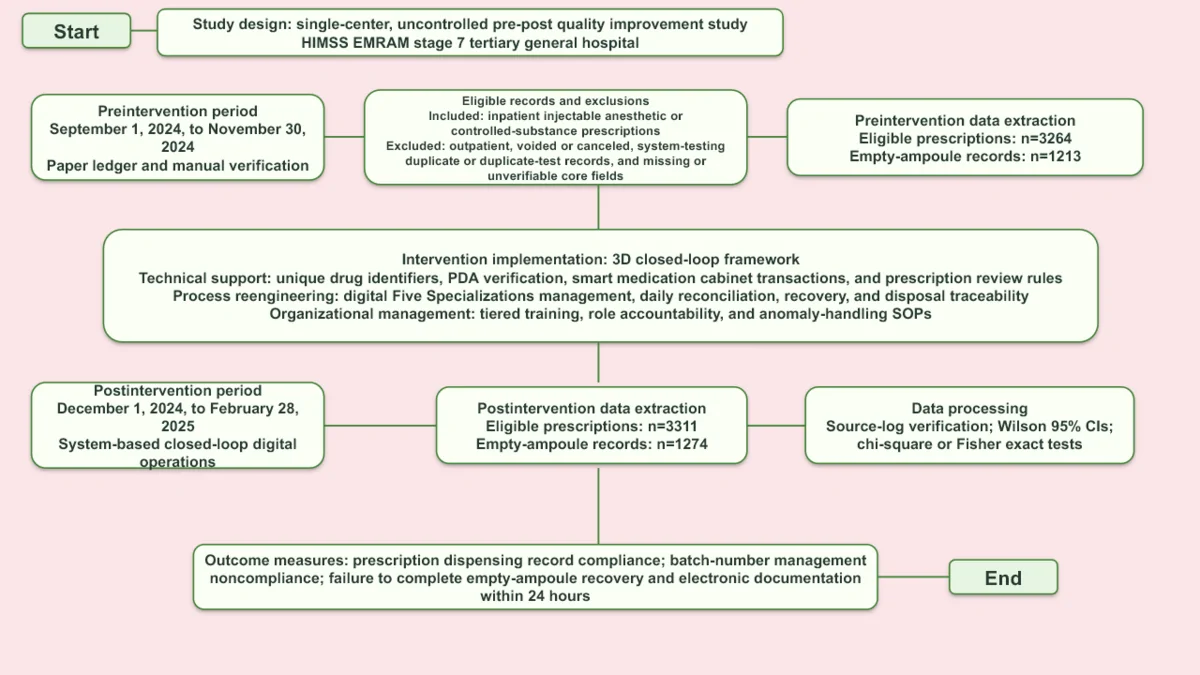
Flowchart of the single-center pre-post quality improvement study. EMRAM: Electronic Medical Record Adoption Model; HIMSS: Healthcare Information and Management Systems Society; PDA: personal digital assistant; SOP: standard operating procedure.

### Ethical Considerations

This retrospective quality improvement study was approved by the ethics committee of Ningbo Hospital of Integrated Traditional Chinese and Western Medicine (Yinzhou District Second Hospital; institutional ethics review, 2026; research approval 076) on August 25, 2026. The study involved a retrospective analysis of deidentified routine operational records generated during routine clinical care before ethics review. No additional patient contact, study-specific intervention, or prospective data collection was undertaken. Individual informed consent was not obtained because only deidentified operational records were analyzed.

### Setting

The study was conducted at a tertiary general hospital that had achieved HIMSS EMRAM stage 7 certification [14]. HIMSS EMRAM stage 7 is the highest maturity stage in this model and indicates that an institution has implemented an advanced digital medical record environment, closed-loop medication-related workflows, and systematic use of electronic data and analytics to support clinical quality improvement [14]. In this context, a tertiary general hospital refers to a high-level referral hospital providing inpatient, surgical, emergency, intensive care, and specialty medical services. During the study period, the hospital had 1150 open beds according to hospital administrative reports, and the analytic dataset included controlled substance prescriptions from 34 active prescribing departments recorded in the prescription review system. The hospital used an integrated hospital information system, anesthesia information system, pharmacy management system, and nursing execution system. The nursing execution system refers to the nurse-facing electronic workflow module used for medication administration documentation and bedside verification. The integrated hospital information system, anesthesia information system, pharmacy management system, and nursing execution system were supplied by Neusoft Corporation. The prescription-review system was an integrated deployment combining Neusoft Corporation with the locally used YiYao prescription-review system. The bedside personal digital assistant (PDA) was a Neusoft Mobile Medical Care PDA manufactured by Neusoft Corporation; no separate numeric model identifier is legible on the available device label. The smart medication cabinets were NuboMed M2300 units manufactured by Shenzhen NuboMed Technology Co, Ltd.

### Participants and Records

The primary analytic unit was the individual prescription dispensing record (line item). Each line item represented 1 ordered medication line and its ordered quantity. For injectable controlled substances, related verification, administration, recovery, and disposal records were linked through the relevant system identifiers when available. The empty-ampoule outcome was analyzed separately at the used-ampoule record level and was not inferred from the ordered quantity of a prescription dispensing record. Multiple line items could belong to the same prescription because of split dosing. Each record was assigned a unique analytic row identifier (row_id) in the cleaned dataset. The prescription identifier (rx_id) linked multiple line items belonging to the same prescription when split dosing generated >1 record.

The empty-ampoule outcome analysis was restricted to eligible used-ampoule records for injectable controlled substances and measured whether recovery and electronic documentation were completed within 24 hours. Because a single injectable prescription could generate >1 used ampoule, this outcome was counted at the ampoule record level rather than the prescription level.

### Intervention

The intervention was a 3D closed-loop framework for controlled substance safety that integrated technical support, process reengineering, and organizational management (Figure 2). The framework used unique drug identification codes (Figure S1 in Multimedia Appendix 1) as the core traceability element and embedded prescription review together with 4 batch-verification checkpoints: dispensing, administration, empty-ampoule return, and daily reconciliation. Final disposal status was documented and reconciled at the fourth checkpoint rather than treated as a separate fifth verification node. The coding rules and 4-node batch-verification logic are provided in Section S1 in Multimedia Appendix 1; the 6 prescription review rule categories in Section S2 in Multimedia Appendix 1; interface fields and offline synchronization logic in Section S3 (Figures S2 and S3) in Multimedia Appendix 1; tiered alerts in Section S4 in Multimedia Appendix 1; and daily reconciliation and anomaly-handling standard operating procedures (SOPs) in Section S5 in Multimedia Appendix 1.

**Figure 2:**
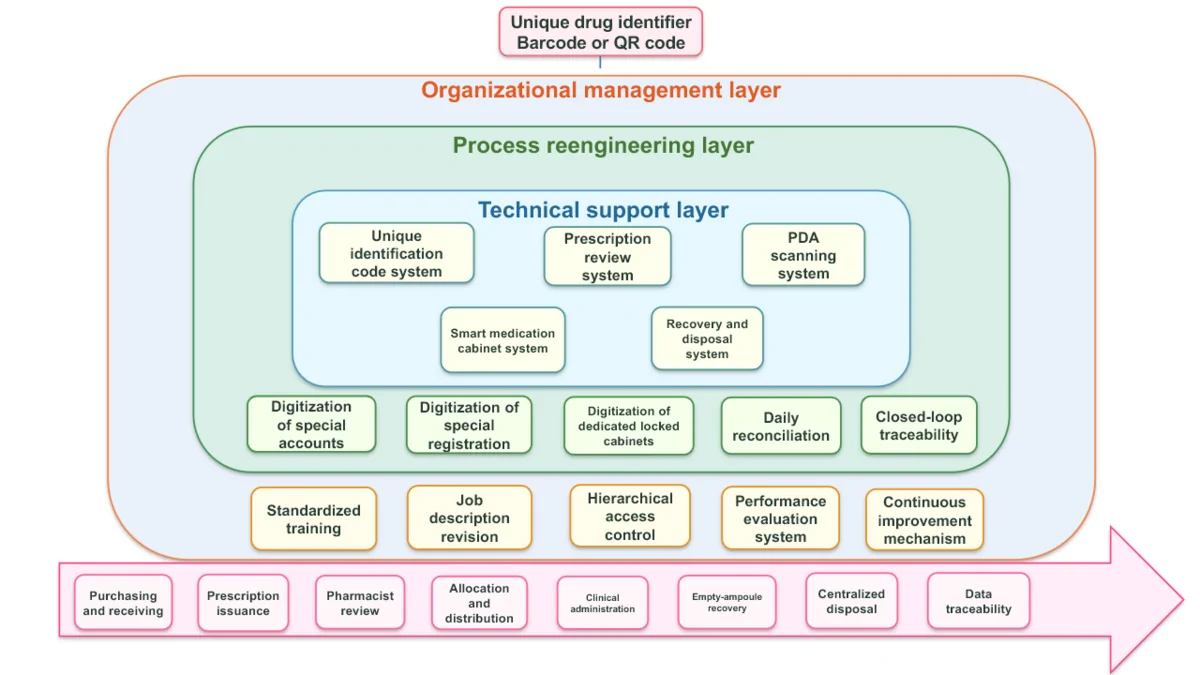
Architecture of the 3D closed-loop framework for controlled substance safety. The framework used the unique drug identification code (Figure S1 in Multimedia Appendix 1) as the core traceability element. The technical, process, and organizational layers jointly supported closed-loop management from inventory receipt to dispensing, administration, empty-ampoule recovery, daily reconciliation, and final disposal documentation. PDA: personal digital assistant.

The intervention evolved in 4 phases. Phase 1, in August 2024, focused on system configuration, drug coding, and hardware installation. Phase 2, during September 2024 and October 2024, used pilot testing in 2 surgical wards and 1 nonsurgical ward to refine the scanning workflow and exception-handling procedures based on frontline feedback. Phase 3, in November 2024, consisted of hospital-wide staff training, including classroom sessions and bedside drills, with role-specific certification for nurses and pharmacists. Phase 4 began on December 1, 2024, when the framework was implemented across all 34 prescribing departments. During medication administration, nurses used PDAs to scan the patient wristband, the active medical order, and the medication identifier before giving the medication; the system then performed triple verification of patient identity, order concordance, and medication identity. During empty-ampoule recovery, pharmacists or nurses used the PDA to scan eligible returned empty ampoules. During the first 2 weeks of full implementation, frontline staff required additional bedside support for scanning and exception handling; this adaptation was addressed through just-in-time pharmacy coaching and the placement of a quick-reference guide at each medication station.

In the prescription review system, indication matching used the primary diagnosis only; high-severity indication mismatches triggered an automatic block, whereas lower-severity mismatches triggered pharmacist review. This indication-mismatch severity ranking was separate from the level 1 to level 3 alert scale used in Section S4 in Multimedia Appendix 1. The pain-assessment rules in Section S2 in Multimedia Appendix 1 applied only to analgesic prescriptions when pain scoring was clinically applicable and were not mandatory criteria for intraoperative anesthetic administration or protocol-driven anesthesia induction or maintenance. The controlled substance rule library and study scope were limited to narcotic drugs and class 1 psychotropic drugs; class 2 psychotropic drugs were outside the analytic scope.

In the process reengineering layer, the “five dedicated” management principles for controlled substances [15], namely dedicated personnel, dedicated lockable cabinets, dedicated ledgers, dedicated prescriptions, and dedicated registration, were digitized into a traceable workflow covering dispensing, administration, empty-ampoule return, daily reconciliation, and final disposal documentation. The final disposal status was captured during daily reconciliation and anomaly closure. Coding rules, 4-node batch verification, interface logic, daily reconciliation, and anomaly-handling SOPs are provided in Sections S1, S3, and S5 in Multimedia Appendix 1.

In the organizational management layer, pharmacists supervised batch-number traceability, audited process compliance, and instructed staff in system operation. Nurses performed the final bedside verification and were responsible for empty-ampoule recovery. Physicians remained responsible for rational prescribing and collaborated with pharmacy and nursing staff when exceptions occurred. Administrators monitored system performance, reviewed process metrics, coordinated optimization, and served as the regulatory liaison. These responsibilities were summarized in Table 1.

**Table 1:** Core responsibilities of staff under the closed-loop controlled substance workflow.

Positions	Traditional roles	Core responsibilities under the new process
Pharmacist	Medication dispenser	Batch-number tracing supervisor, process compliance auditor, and system operation instructor
Nurse	Order executor	Bedside final verifier and responsible for empty-ampoule recovery
Physician	Prescriber	Person responsible for rational drug use and collaborator in process compliance
Administrator	Daily supervisor	System backend monitor, metrics analyst, process optimization champion, and regulatory liaison

### Outcomes

Batch-number management was evaluated separately using a simple random sample of 1000 unique eligible prescriptions from each period after duplicate and eligibility checks. This audit had a prescription-level denominator and was distinct from the prescription dispensing record denominator used for the primary compliance outcome. For medication lines with ordered quantities greater than 1, the same record could represent multiple tablets, patches, or injection units, and the linked batch-number and recovery or disposal logs therefore reflected the ordered quantity rather than a single physical unit. The operational definitions and calculation methods for each indicator are presented in Table 2.

**Table 2:** Operational definitions and calculation methods for each indicator.

Indicators	Calculation method	Judgment criteria (with authoritative basis)
Prescription dispensing record compliance rate	(Number of compliant prescription dispensing records/total number of included prescription dispensing records)×100% (95% CI)	Appropriate indications: consistent with the hospital prescription review rule library and International Classification of Diseases, 10th Revision eligibility rules used during the study period (Section S2 in Multimedia Appendix 1) Correct dosage: not exceeding the maximum dosage specified in the product insert or the institution-specific decision-support threshold No drug-drug interactions: in accordance with the hospital prescription review and interaction-alert rules Qualified prescribing authority: physicians with hospital-approved authorization to prescribe narcotic and class 1 psychotropic drugs [15]
Batch-number management noncompliance rate in the prescription audit sample	(Number of noncompliant prescriptions in the 1000-prescription audit sample/1000 audited prescriptions)×100% (95% CI)	Mismatched batch numbers between the prescription and dispensed drugs Incomplete batch-number records in electronic ledgers Untraceable batch numbers during empty-ampoule recovery
Rate of failure to complete empty-ampoule recovery and electronic documentation within 24 hours	(Number of used ampoules without completed recovery and electronic documentation within 24 hours/total number of used anesthetic drug ampoules)×100% (95% CI)	Failure to complete empty-ampoule recovery and electronic registration within 24 hours after drug use. Final disposal was performed once daily with 2-person verification and separate electronic closure in the same system.

### Statistical Analysis

Two data extractors independently extracted the operational records and cross-checked them against the source documents. No record in the final source-verified dataset was altered; flagged records were either retained after source confirmation or excluded before outcome computation.

Interrater reliability was assessed using a prespecified stratified random sample of 500 records per outcome domain from the source-verified analytic dataset (n=250 preintervention and n=250 postintervention records per domain). Two reviewers independently classified each sampled record without access to each other’s ratings. Cohen κ was calculated before adjudication from the 2 independent rating columns using the standard 2-by-2 agreement-table formula. The κ coefficients indicated high agreement: prescription dispensing record compliance, 0.898; batch-number management, 0.956; and empty-ampoule recovery, 0.964. Discrepant reviewer ratings were retained in the κ calculation and marked for adjudication in the audit dataset; the final adjudication or status column was not used to compute κ. The κ summary is provided in Table S4 in the Multimedia Appendix 1. The primary outcome analysis used the final source-verified analytic dataset. Detailed data-quality verification and sensitivity analysis results are provided in Table S2 in the Multimedia Appendix 1. As a leave-2-records-out sensitivity analysis, the prescription dispensing record–compliance outcome was recalculated after excluding the 2 source-confirmed preintervention period flagged records, reducing the preintervention period denominator from 3264 to 3262.

### Contextual and Confounding Factors

To contextualize the observation windows, we summarized surgical workload descriptively using surgical patient counts based on unique admission numbers. The preintervention and postintervention periods included 4837 and 4191 unique surgical admissions, respectively. Table S1 in Multimedia Appendix 1 also reports bed occupancy, open beds, staffing levels, prescribing-department counts, controlled substance prescription volume, and the prescription share of the 5 highest-volume departments. These indicators were used as descriptive context only and do not establish equivalence between periods or eliminate residual temporal confounding.

The same hospital information system, anesthesia information system, pharmacy management system, and nursing execution system were used during both periods, and no major platform upgrade was performed during the observation windows. Training and operational drills were completed before implementation; however, residual temporal confounding from holiday periods, staffing patterns, workload variation, and unmeasured case-mix differences could not be fully eliminated. Exploratory subgroup analyses were conducted by drug type when reliable denominators were available. Department-type subgroup estimates were not retained because a matched deidentified denominator was not available. No formal interaction test was performed, and subgroup results were interpreted descriptively.

## Results

### Study Sample and Context

The barcode-enabled closed-loop framework was implemented across the controlled substance medication-use workflow and generated stable operational logs during the postintervention period. The analysis included 3264 prescription dispensing records before implementation and 3311 prescription dispensing records after implementation.

The 2 observation windows included 4837 and 4191 unique surgical admissions, respectively. Table S1 in Multimedia Appendix 1 summarizes bed occupancy rates, open beds, prescribing departments, staffing, controlled substance prescription volume, and the prescription share of the 5 highest-volume departments. These data provide descriptive context only and do not establish equivalence between the periods. Residual confounding from the New Year and Spring Festival holiday periods cannot be fully excluded.

### Prescription Dispensing Record Compliance

The total number of prescription dispensing records was 3264 before the intervention and 3311 after the intervention. Prescription dispensing record compliance increased from 94.82% (3095/3264) to 98.7% (3268/3311), while noncompliance decreased from 5.18% (169/3264) to 1.3% (43/3311). The difference was statistically significant (*χ*^2^_1_=79.26; *P*<.001), as shown in Table 3.

**Table 3:** Comparison of prescription dispensing record compliance rates.

Indicators	Preintervention period (n=3264), n (%; 95% CI)	Postintervention period (n=3311), n (%; 95% CI)	*χ*^2^ test (*df*)	*P* value
Compliance	3095 (94.82; 94.01-95.53)	3268 (98.7; 98.26-99.03)	79.26 (1)	<.001
Noncompliance	169 (5.18; 4.47-5.99)	43 (1.3; 0.97-1.74)	79.26 (1)	<.001

### Batch-Number Management

Using the same prescription-level audit frame of 1000 unique eligible prescriptions per period, the overall batch-number management noncompliance rate decreased from 2.4% (24/1000) to 0.6% (6/1000; *χ*^2^_1_=10.96; *P*=.001). Among the 3 nonmutually exclusive subcategories, prescription batch-number mismatch and incomplete electronic ledger records decreased significantly in Fisher exact tests, whereas batch-number mismatch during empty-ampoule recovery decreased in the same direction but did not reach statistical significance (*P*=.18; Table 4).

**Table 4:** Comparison of batch-number management noncompliance.

Indicators^a^	Preintervention period (n=1000)	Postintervention period (n=1000)	*P* value^b^
Prescription batch-number mismatch, n (%)	12 (1.2)	3 (0.3)	.03
Incomplete electronic ledger records, n (%)	12 (1.2)	3 (0.3)	.03
Batch-number mismatch in empty-ampoule recovery, n (%)	7 (0.7)	2 (0.2)	.18
Overall noncompliance rate, n (%; 95% CI)	24 (2.4; 1.62-3.55)	6 (0.6; 0.28-1.3)	.001

^a^The 3 subcategories were not mutually exclusive; a prescription could be counted in >1 category.

^b^The *P* values for the 3 individual noncompliance indicators were calculated using the Fisher exact test. The *P* value for the overall noncompliance rate was calculated using the chi-square test (*χ*^2^_1_=10.96).

### Empty-Ampoule Recovery and Electronic Documentation Within 24 Hours

A total of 1213 used ampoules at baseline and 1274 postintervention used ampoules were analyzed. The rate of failure to complete empty-ampoule recovery and electronic documentation within 24 hours decreased from 5.61% (68/1213) to 0.78% (10/1274), a statistically significant difference (*χ*^2^_1_=47.54; *P*<.001; Table 5). Final disposal was performed once daily with 2-person verification and electronic closure in the same system, but disposal completion was not part of the 24-hour outcome denominator.

**Table 5:** Comparison of empty-ampoule recovery and electronic documentation within 24 hours before and after implementation.

Indicators	Preintervention period (n=1213), n (%; 95% CI)	Postintervention period (n=1274), n (%; 95% CI)	*χ*^2^ test (*df*)	*P* value
Rate of failure to complete recovery and electronic documentation within 24 hours	68 (5.61, 4.45-7.05)	10 (0.78; 0.43-1.44)	47.54 (1)	<.001
Completed recovery and electronic documentation within 24 hours rate	1145 (94.39, 92.95-95.55)	1264 (99.22; 98.56-99.57)	47.54 (1)	<.001

### Implementation Fidelity

Postintervention implementation fidelity was high according to operational system logs (Table 6). System stability was high: system uptime was 99.92% (30,216/30,240) monitored node-hours, calculated as 14 monitored nodes×90 days×24 hours and covering the full 90-day postintervention period, and smart medication cabinet network uptime was 99.88% (25,889/25,920) monitored cabinet-hours, calculated as 12 monitored cabinets×90 days×24 hours and covering the full 90-day postintervention period. Operational compliance was also high: PDA verification execution rate was 99.76% (3303/3311) of eligible medication administration verification events, with each event comprising wristband, order, and medication-ID verification. Medical order scan execution was 99.64% (3299/3311), wristband scan verification was 99.82% (3305/3311), drug identifier code scanning was 99.79% (3304/3311), empty-ampoule scanning within 24 hours was 99.22% (1264/1274), and abnormal-incident closure was 100% (52/52). Network downtime was counted as an uptime failure even when the local cache remained available, because the cabinet was not fully connected to the live system. These high rates reflect the system-enforced workflow design: required scans were embedded as mandatory steps before medication administration, dispensing, empty-ampoule recovery, or incident closure could be completed, and scan failures generated hard stops or exception logs rather than relying only on voluntary manual compliance. These indicators were organized to reflect multiple dimensions of implementation fidelity, including system stability, operational adherence to required workflow steps, data synchronization completeness, and anomaly-process closure [16].

**Table 6:** Core metrics for framework implementation fidelity.

Indicators			Result, n/N (%)		Data source
**System stability**					
	System uptime (monitored node-hours)	30,216/30,240 (99.92)		System operation log	
	Smart medication cabinet network uptime (monitored cabinet-hours)	25,889/25,920 (99.88)		Cabinet network log	
	Data synchronization integrity rate during network outages	320/320 (100)		Offline synchronization log	
**Operational compliance^a^**					
	PDA^b^ verification execution rate	3303/3311 (99.76)		PDA scan log	
	Medical order scan execution rate	3299/3311 (99.64)		Medical order scan execution log	
	Wristband scan verification rate	3305/3311 (99.82)		Wristband scan log	
	Scanning rate of drug identification codes	3304/3311 (99.79)		Drug identifier scan log	
	Empty-ampoule scanning rate within 24 hours	1264/1274 (99.22)		Empty-ampoule recovery scan log	
Anomaly process: closure rate for abnormal incidents			52/52 (100)		Incident closure log

^a^For the operational-compliance indicators, the denominator was the number of eligible postintervention medication administration verification events (N=3311), unless otherwise specified. Each verification event required the wristband, medical order, and medication identifier scans. The empty-ampoule indicator used a separate denominator based on eligible used-ampoule records (N=1274).

^b^PDA: personal digital assistant.

### Sensitivity and Subgroup Analyses

Four source-verified prescription dispensing records were flagged during source verification. Two preintervention period records were source-confirmed and retained in the primary analysis; 1 postintervention period duplicate record was excluded to avoid double counting, and 1 postintervention period incomplete record was excluded because required fields were missing. In the leave-2-records-out sensitivity analysis, prescription dispensing record compliance was 94.82% (3093/3262) before implementation and 98.7% (3268/3311) after implementation, with the between-period comparison remaining statistically significant (*P*<.001). The batch-number and 24-hour empty-ampoule outcomes were unchanged after source verification. Exploratory prescription dispensing record–level descriptive subgroup results are shown in Table S3 in Multimedia Appendix 1; no formal interaction test was performed.

## Discussion

### Principal Findings

This single-center pre-post quality improvement study implemented a 3D closed-loop framework for controlled substance safety in a HIMSS EMRAM stage 7 hospital [10,16-18]. After implementation, 3 operational process indicators changed in a favorable direction: prescription dispensing record compliance, batch-number management, and 24-hour empty-ampoule recovery and electronic documentation. These findings suggest that routinely generated system logs and medication management records can be used to quantify the implementation of a closed-loop controlled substance workflow in a digitally mature hospital [10,16-18].

### Comparison With Prior Work

Prior work on medication safety has often evaluated individual components, such as barcode medication administration, smart medication cabinets, electronic prescribing checks, or access-control rules [17,19-21]. These approaches are important but usually focus on a single stage of the medication-use process. The present framework differs by connecting several existing regulatory and best-practice requirements into an interlocked operational workflow. Rather than treating prescription review, batch-number documentation, cabinet access, bedside verification, empty-ampoule recovery, and anomaly handling as separate tasks, the framework links these tasks through shared identifiers, system-enforced checkpoints, and accountable roles.

The contribution of this study is therefore not that batch-number documentation, empty-ampoule recovery, prescription authority, or controlled substance accounting are new requirements. These are already expected in regulated medication management practice and are consistent with professional guidance on preventing controlled substance diversion [22]. The added value is the systematization of these requirements into a traceable and measurable digital process [8-10,16-18]. The framework makes the relationship between the physical medication, the electronic order, the batch number, the user action, and the final recovery or disposal record more explicit than in a paper ledger or a single-node electronic module [8-10,17,18]. National or institutional narcotics-management information systems have also shown the value of structured life cycle monitoring for medical narcotics [18]. In contrast to single-module optimization, the framework provides an integrated technology-process-organization model and discloses implementation parameters for coding, prescription rule interception, interface synchronization, tiered accountability, and anomaly SOPs [8-10,16-18].

### Interpretation and Implications

End-to-end batch-number traceability may be an important component of the observed improvement in 24-hour empty-ampoule recovery and electronic documentation because it links the dispensing record, administration verification, returned ampoule, 24-hour recovery and documentation record, daily reconciliation record, and final disposal status. However, this component was implemented as part of a bundled intervention that also included prescription review rules, smart medication cabinets, PDA-based verification, staff training, role redefinition, daily reconciliation, and anomaly-handling SOPs. The study design cannot isolate the independent contribution of any single component. The most cautious interpretation is that batch-number traceability was a plausible contributing component within a broader sociotechnical intervention. The apparent effect of hard blocking may also reflect a short adaptation period in which frontline nurses and pharmacists adjusted to more explicit scanning and exception-handling routines. For hospitals with advanced digital maturity, the framework may offer a practical implementation reference; for hospitals with less integrated information systems, phased adaptation and local feasibility testing would be necessary [8-11,16-20].

### Limitations

The principal limitation is that this was a single-hospital pre-post study without randomization or a concurrent control group, so the findings support temporal association rather than causal inference.

Several factors limit the generalizability of our findings. First, the study was conducted in a HIMSS EMRAM stage 7 hospital with integrated hospital information systems, PDA workflows, and smart medication cabinet support, so transferability to hospitals with lower digital maturity may be limited [4,5,10,16-18,20]. However, not all components of the framework require mature information systems: the organizational management layer, including role redefinition, daily reconciliation, and anomaly-handling SOPs, can be implemented in hospitals with basic IT infrastructure and then expanded stepwise as technical capacity improves [8-11,16-18]. Second, the study focused on process indicators rather than patient-level clinical outcomes, so whether the observed improvements translate into fewer diversion events, fewer adverse drug events, or better patient outcomes remains to be established. Potential unintended consequences, including additional scanning time, workflow burden, alert fatigue, and attempts to bypass system controls, were not systematically measured. Therefore, the absence of reported major unintended consequences should not be interpreted as evidence that such effects did not occur. Implementation costs, staff time, maintenance requirements, and opportunity costs were not prospectively itemized, so no formal cost or cost-effectiveness analysis was performed. Third, the single-center uncontrolled pre-post design limits external validity and precludes causal inference. Finally, the observation window included the New Year and Spring Festival holidays, which may have affected staffing patterns and workload in ways that this workload indicator alone cannot fully capture. Sustainability was not formally evaluated, and continued rule maintenance, periodic refresher training, and local governance will be needed to preserve performance as workflows evolve.

### Conclusions

This single-center pre-post study showed that a 3D closed-loop framework integrating technical traceability, process redesign, and organizational accountability was temporally associated with improved controlled substance process indicators in a HIMSS EMRAM stage 7 hospital.

Although the full framework assumes mature digital infrastructure, its components can be introduced incrementally. Hospitals with less integrated information systems may first strengthen the organizational management layer, including role redefinition, daily reconciliation, and anomaly-handling SOPs, and then progressively add technical supports such as PDA-based verification and smart medication cabinets. Because the framework relies on routinely generated operational logs, it may offer a practical quality improvement path even where digital maturity is limited.

Future multicenter studies with stronger designs and longer follow-up are needed to test sustainability, transferability, and effects on patient-level outcomes and controlled substance diversion events.

## Appendix

Multimedia Appendix 1 Supplementary methods, implementation details, data quality checks, subgroup analyses, and supporting tables and figures.

## Abbreviations

EMRAM: Electronic Medical Record Adoption Model
HIMSS: Healthcare Information and Management Systems Society
iCHECK-DH: Guidelines and Checklist for the Reporting on Digital Health Implementations
PDA: personal digital assistant
SOP: standard operating procedure
SQUIRE 2.0: Standards for Quality Improvement Reporting Excellence

## Data Availability

Deidentified aggregate data supporting the findings are available from the corresponding author upon reasonable written request. Requests will undergo formal review by the hospital data security committee before any data are released. Individual-level operational records cannot be shared publicly because of patient confidentiality and institutional data-governance requirements.
