# Supplementary material for: A Quantifiable 3D Closed-Loop Framework for Controlled Substance Safety in a HIMSS Electronic Medical Record Adoption Model (EMRAM) Stage 7 Hospital: Pre-Post Quality Improvement Study
Source: JMIR Med Inform. 2026 Sep 25;14:e98247. doi: 10.2196/98247 (PMC13653207; doi:10.2196/98247)
Supplement: Supplementary file 1 [file medinform_v14i1e98247_app1.pdf]

Multimedia Appendix. Supplementary implementation materials and baseline context

Item	Title	Manuscript location
Section S1	Rules for assigning unique drug identifiers and labeling examples	Methods: Intervention
Section S2	Six categories of prescription-review and interception rules	Methods: Intervention
Section S3	Interface structure for smart medicine cabinets, PDAs, and HIS	Methods: Intervention
Section S4	Tiered alert mechanism	Methods: Intervention
Section S5	Daily reconciliation checklist and anomaly-handling SOP	Methods: Intervention
Table S1	Contextual workload and staffing indicators during the pre- and post-intervention periods	Methods: Contextual and Confounding Factors; Results: Study Sample and Context
Table S2	Data-quality verification and sensitivity analyses	Methods: Statistical Analysis; Results: Sensitivity and Subgroup Analyses
Table S3	Exploratory drug-type subgroup analyses at the prescription-dispensing record level	Methods: Statistical Analysis; Results: Sensitivity and Subgroup Analyses
Table S4	Inter-rater reliability based on independent dual-reviewer ratings	Methods: Statistical Analysis

Section S1. Rules for assigning unique drug identifiers and labeling examples

Coding scheme. Dual coding using a one-dimensional barcode (Code 128) and a two-dimensional barcode (QR Code). The QR Code contains the following 9 fields:

Field Number	Field Name	Example	Description
1	National Drug Electronic Supervision Code	86912345678901	Illustrative numeric identifier; not a live production code
2	Unique Hospital Traceability Code	NARC-20240901-00123	Format: NARC-YYYYMMDD-Serial Number
3	Generic Name	Morphine Hydrochloride Injection	Chinese/English
4	Strength	10 mg:1 mL	Dosage and Administration
5	Lot Number	230815A	Must match the batch number on the outer packaging
6	Expiration Date	2025-08-31	Format YYYY-MM-DD
7	Manufacturer	XX Pharmaceutical Co., Ltd.	Standard Company Name
8	Date Received	September 1, 2024	Pharmacy Receipt Date
9	Storage Location	Smart Medicine Cabinet-Cabinet No. 3 - 2nd Shelf	Physical Location Code

Batch-number mandatory association logic (Four Verification Checkpoints)

Bound at Generation: After scanning by the pharmacy system, the original drug batch number is automatically read and entered into the field; manual modification is not permitted.

At the first checkpoint, dispensing, the pharmacist scans the prescription and the medication identifier before cabinet release. The system compares the medication batch number with the HIS dispensing record and stores the result in the dispensing and smart-cabinet transaction logs. If the batch number does not match, the cabinet locks with a red indicator and the discrepancy requires dual verification before correction.

At the second checkpoint, administration, the nurse scans the patient wristband, active medical order, and medication identifier with a PDA before administration. The system compares the scanned batch number with the prescription and dispensing records and stores the result in the nursing execution/PDA administration log. If the batch number or patient-order match fails, administration is blocked and an exception log is generated.

At the third checkpoint, empty-ampoule return, when empty ampoules are returned, the nurse or pharmacist scans the ampoule identifier. The system verifies whether the batch number has a corresponding "Used" administration record and stores the result in the empty-ampoule return log. If no corresponding used record exists, the return fails and requires anomaly handling.

At the fourth checkpoint, daily reconciliation and disposal-status documentation, the pharmacist or manager compares dispensed batch numbers, administration records, returned ampoule records, and final disposal status. The system generates a discrepancy or closure report and stores the final disposal status in the reconciliation/anomaly closure record. Batch numbers with unresolved status, such as dispensed but not returned or returned but not closed, require written explanation and closure before monthly summary.

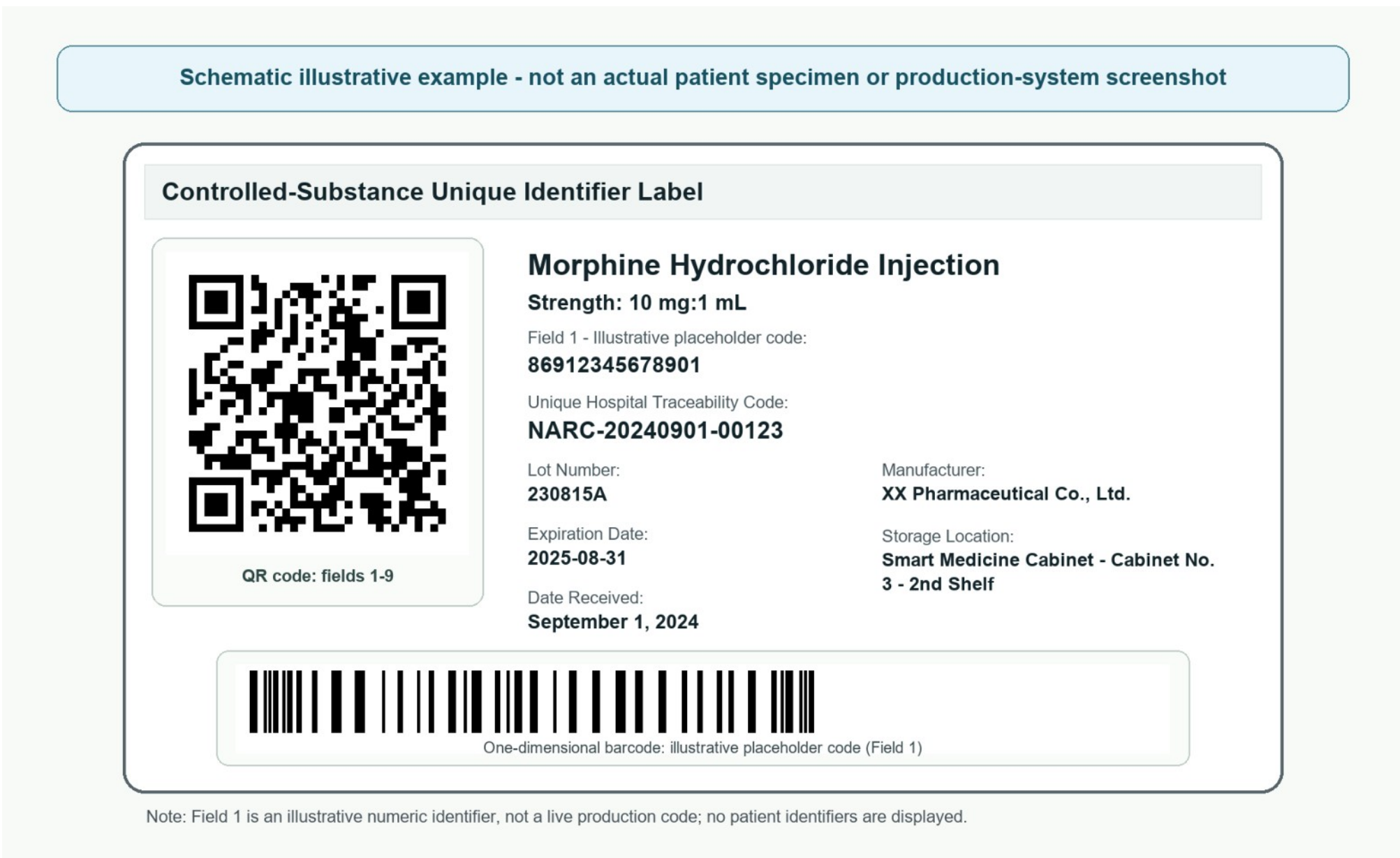

Figure S1. Drug identifier label example. English schematic rendering of the controlled-substance unique identifier label (label-like example; not an actual patient specimen or production-system screenshot). The label displays the nine coded fields defined in the coding-scheme table above (Fields 1-9), with the QR code encoding Fields 1-9 and the one-dimensional barcode corresponding to Field 1 (an illustrative numeric identifier, not a live production code). The example drug

(Morphine Hydrochloride Injection, 10 mg:1 mL) reflects the injectable controlled substances included in this study. No patient identifiers are displayed.

Section S2. Six categories of prescription-review and interception rules

Rule-source note: Thresholds and intercept rules below are traced to one of the following source types: product labeling (study-period package insert or approved labeling), named national or professional guidance used only when directly applicable and formally cited (for example, ASHP guidance on preventing diversion of controlled substances [21]), the hospital International Classification of Diseases, 10th Revision (ICD-10) coding dictionary used during the 2024-2025 study period, the hospital narcotic and Class I psychotropic prescribing authority policy (local implementation based on YWYX-011 and national regulation [15]), the hospital off-label-use approval policy, or locally approved decision-support rules approved by the hospital pharmacy and medical-governance teams. The study scope was limited to narcotic drugs and Class I psychotropic drugs; Class II psychotropic drugs were not included. Institution-specific thresholds were used to trigger clinical review rather than as absolute therapeutic limits.

Scope note: This section describes the prescription-review rule library implemented in the hospital controlled-substance management system. The manuscript scope was limited to narcotic drugs and Class I psychotropic drugs; Class II psychotropic drugs were not included. The prescription-dispensing record-compliance analysis reported in the manuscript included eligible inpatient controlled-substance prescriptions captured by the prescription-review system, including injectable and noninjectable formulations such as fentanyl transdermal patches. The empty-ampoule outcome analysis was restricted to eligible used ampoule records for injectable controlled substances and measured whether recovery and electronic documentation were completed within 24 hours. Therefore, fentanyl transdermal patch rules are included here to document system functionality and reproducible implementation parameters, but fentanyl patches did not contribute to the empty-ampoule recovery/documentation denominator.

Dosage Control Rules

Each rule row lists its source basis in the Source column.

Rule Design Philosophy

Dose control employs a risk-tiered + rationality review model rather than simple, absolute dose blocking. Since opioid medications have no theoretical upper dose limit, clinical practice requires a comprehensive assessment based on patient tolerance, pain intensity, and the severity of surgical stimulation in clinically applicable analgesic settings. The blocking rules focus on identifying dangerous doses and conducting rationality reviews.

Trigger Conditions

A warning is triggered when the prescribed dose exceeds any of the following thresholds in clinically applicable opioid analgesic prescriptions, requiring the physician to confirm its rationality:

Drug	Dose Threshold	Source	Description
Morphine Injection (for non-tolerant patients)	Single dose >15 mg or cumulative dose >60 mg within 24 hours	Rule source: hospital analgesic management policy (104-P-138; issued 2024-11-08) and a study-period institution-specific decision-support threshold approved by the hospital pharmacy and medical-governance teams (used as a clinical-review trigger rather than an absolute therapeutic limit)	Based on the goal-directed analgesia principle (NRS < 4); tolerance and risk of respiratory depression were assessed if this range was exceeded
Morphine Injection (Tolerant Patients)	Single dose >30 mg or 24-hour cumulative dose >120 mg	Rule source: hospital analgesic management policy (104-P-138; issued 2024-11-08) and a study-period institution-specific decision-support threshold approved by the hospital pharmacy and medical-governance teams (used as a clinical-review trigger rather than an absolute therapeutic limit)	Patients with established tolerance must have a documented history of use (continuous use for ≥1 week or daily dose ≥60 mg oral morphine equivalent); dosing beyond these limits requires expert review
Fentanyl transdermal patch	Initial dose >25 μg/h	Rule source: fentanyl transdermal patch prescribing information (study-period version)	The initial dose is typically 12 μg/h or 25 μg/h; higher doses require consultation with a pain specialist or anesthesiologist
Sufentanil (intraoperative)	Single dose >10 μg/kg	Rule source: hospital analgesic management policy (104-P-138;	High doses (>8 μg/kg) were indicated only for highly stimulating

Drug	Dose Threshold	Source	Description
		issued 2024-11-08) and sufentanil prescribing information (study-period version)	scenarios such as major cardiac surgery; the type of surgery was specified

Additional Safety Requirements

Respiratory depression monitoring: For opioid analgesic prescriptions exceeding the threshold in clinically applicable settings, the system automatically displays a warning: "Opioid prescription exceeding the threshold. Ensure respiratory support is ready (standby naloxone, oxygen supply), and closely monitor SpO_2, RR, and sedation scores after administration."

Naloxone Availability: Ensure naloxone is immediately accessible prior to administration to address potential respiratory depression.

For analgesic prescriptions in clinically applicable settings, prescriptions exceeding the threshold included a clinical justification (e.g., "Pain NRS ≥ 7, well tolerated" or "Anticipated high stimulation following major cardiac surgery"), which the system archived for future reference. Pain intensity was scored using the numeric rating scale (NRS).

Intervention Measures

Level 1 interception (mild threshold exceedance): A yellow pop-up window displays the message: "Prescribed dose exceeds the standard range; please confirm clinical justification and provide a reason." The prescription may be submitted but will be logged.

Level 2 interception (severe threshold exceedance): A red pop-up window displayed the message: "Dose significantly exceeds safe limits; please perform a two-person review and document the clinical rationale." The employee ID of the reviewing pharmacist or supervising physician was entered before submission, and the system automatically forwarded the request to the Pharmacy Department.

Patient Education: For analgesic prescriptions exceeding thresholds (especially fentanyl patches), the system automatically pushes patient education information, emphasizing usage precautions (such as avoiding contact with heat sources and patch replacement intervals).

Indication Matching Rules

Rule source: hospital ICD-10 coding dictionary used during the 2024-2025 study period (study-period version) and an institution-specific decision-support rule; ICD-10 labels were verified against that dictionary version before reuse.

Rule Design Philosophy

A three-tier review strategy combining primary-diagnosis matching, pain severity assessment, and a "reasonable use" whitelist is adopted rather than a simple ICD-10 match. The system used the primary diagnosis only; high-severity indication mismatches triggered an automatic block, whereas lower-severity mismatches triggered pharmacist review. This indication-mismatch severity ranking was separate from the Level 1-3 alert scale used in Section S4. The pain-severity rules below applied only when pain scoring was clinically applicable. An off-label approval channel is retained to ensure rational drug use in clinically necessary situations.

Trigger Conditions

A warning is triggered when the prescribed medication has no clinical relevance to the patient's primary diagnosis, or does not meet the criteria in the following indications whitelist, and the pain intensity assessment (NRS score) is mild to moderate (<4 points) in clinically applicable analgesic settings.

Drug	Permitted Primary Indications (primary diagnosis only; ICD-10 Code Reference)	Source
Morphine Injection	Management of moderate to severe pain (primary diagnosis only): R52 (Pain), Z51.1 (Chemotherapy session for neoplasm), Z51.5 (Palliative care) (verified against the hospital ICD-10 coding dictionary used during the 2024-2025 study period)<br>Ischemic chest pain due to acute coronary syndrome: I20-I25 (Ischemic heart disease)<br>Aortic dissection: I71.0–I71.1<br>Postoperative analgesia: Z98.8 (Postoperative conditions)<br>Pain in advanced cancer: C00-C97 (Malignant neoplasms)	Rule source: hospital ICD-10 coding dictionary used during the 2024-2025 study period (study-period version) and an institution-specific decision-support rule
Fentanyl (injection)	Sedation and analgesia before, during, and after anesthesia (combined anesthesia)<br>Various moderate to severe pain: R52<br>Short-acting, potent analgesia (surgical analgesia)	fentanyl injection prescribing information

Drug	Permitted Primary Indications (primary diagnosis only; ICD-10 Code Reference)	Source
	Postoperative analgesia: Z98.8	
Fentanyl Transdermal Patch	Chronic moderate-to-severe pain (requiring continuous use of opioid analgesics)<br>Cancer pain: C00–C97<br>Chronic non-cancer pain (with an established treatment regimen)	fentanyl transdermal patch prescribing information (study-period version) and a locally approved decision-support rule
Sufentanil	Anesthetic analgesia during surgery<br>Postoperative analgesia<br>Anesthetic induction and maintenance	sufentanil prescribing information (study-period version) and a study-period institution-specific decision-support rule approved by the hospital pharmacy and medical-governance teams
Pethidine	Pre-anesthetic administration<br>Postoperative analgesia<br>Treatment of shivering (R25.2)<br>Moderate to severe pain (short-term use; use with caution in elderly patients)	institution-specific decision-support rule approved by the hospital pharmacy and medical-governance teams

Pain intensity assessment (NRS score)

Scope note: These pain-assessment rules applied to opioid analgesic prescriptions in routine clinical dispensing workflows when pain scoring was clinically applicable. They were not mandatory criteria for intraoperative anesthetic administration or protocol-driven anesthesia induction/maintenance; when patients could not self-report, the Behavioral Pain Scale (BPS) could be used instead of the NRS when clinically appropriate.

For opioid analgesic prescriptions where pain scoring was clinically applicable, the NRS score (or the BPS score for patients unable to self-report) was documented. The assessment results served as supplementary evidence for determining the appropriateness of the prescription.

NRS 1–3 (mild, clinically applicable analgesic settings): Opioids alone were generally not recommended; non-opioid medications or physical therapy were preferred.

NRS 4–6 (moderate, clinically applicable analgesic settings): Opioids may be considered, typically with multimodal analgesia.

NRS 7–10 (severe, clinically applicable analgesic settings): Opioid analgesia may be appropriate when severe pain is documented; specific drug choice and dose should follow the treating team's clinical judgment and the applicable workflow.

Management of Special Cases

When not indicated by diagnosis but clinically necessary, the prescription must go through the off-label approval process described in the Approval Rules for Off-Label Use section below; may be prescribed only after approval.

Emergency situations (Emergency Department/intensive care unit (ICU), when pain scoring was clinically feasible): For pain NRS ≥ 7 or clinically urgent conditions (e.g., aortic dissection, acute myocardial infarction), a single dose may be prescribed temporarily; if pain scoring was not feasible, the prescriber documented the clinical rationale and completed the approval record in the hospital information system within 24 hours.

Patient Education: When prescribing a fentanyl transdermal patch for the first time, the system automatically sends patient education materials (emphasizing the 72-hour replacement cycle, avoiding contact with heat sources, and proper storage of the patch).

Interception Actions

A pop-up window prompts: "Indications do not match the drug's intended use. Please confirm or submit a request through the off-label approval channel."

The system automatically generates a pending approval request and forwards it to the Pharmacy Department's online approval system.

The prescription cannot be submitted until approval is complete.

Duplicate Prescription Rules

Rule source: institution-specific decision-support rule aligned with Anatomical Therapeutic Chemical (ATC) code-based medication-class grouping.

Trigger condition: Prescribing two or more anesthetic drugs with the same mechanism of action to the same patient within the same consultation period (within 24 hours).

Identification Logic: Based on the fourth digit of the ATC code being the same (e.g., N02AB refers to phenylpiperidine derivatives, including fentanyl, sufentanil, and remifentanil).

Interception Action: Displays the prompt "Duplicate prescription of the same class of anesthetic drugs; please delete one of them" and prohibits submission.

Drug Interaction Rule

Rule source: institution-specific decision-support rule informed by the hospital-approved drug-interaction reference set (study-period version).

Trigger Condition: A known drug interaction exists between the controlled substance in the prescription and other medications the patient is currently taking.

Contraindication Examples:

Morphine + Monoamine oxidase inhibitors (e.g., selegiline): Severe central nervous system depression

Fentanyl + Ritonavir: Risk of increased blood concentration

Pethidine + Promethazine: Superimposed respiratory depression

Rule source: institution-specific decision-support rule informed by the hospital-approved drug-interaction reference set (study-period version).

Prescription Authority Control Rules

Rule source: hospital narcotic and Class I psychotropic prescribing authority policy (version used during the study period), which operationalized the national narcotic and psychotropic management regulation [15].

Trigger Conditions:

The prescribing physician lacks prescribing authority for narcotic and Class I psychotropic drugs at this hospital;

Prescribing beyond one's authorized level (e.g., a resident prescribing a red prescription without supervision from a senior physician);

Prescribing authority has expired or been suspended.

The authorization levels are as follows:

Resident Physician: May only prescribe a single dose (24 hours) with authorization from a senior physician

Attending Physician: May prescribe a 3-day supply

Associate Chief Physician/Chief Physician: May prescribe a 7-day supply (for cancer pain/chronic pain)

Interception Action: Displays a prompt stating "No authorization to prescribe narcotic or Class I psychotropic drugs" or "Prescription exceeds authorized level," and locks the prescription button.

Approval Rules for Off-Label Use

Rule source: hospital off-label-use approval policy (version used during the study period).

Trigger Conditions: The prescription does not comply with indication matching rules or dosage rules, but the physician deems it clinically necessary.

The process is as follows:

The physician clicks "Request Off-Label Use" in the HIS;

Enter the rationale (clinical basis; PDF literature can be uploaded);

The system forwards the request to the Director of Pharmacy and the Ethics Committee for online approval;

Upon approval, the system temporarily unlocks the prescription (valid for 24 hours);

All approval records are archived in the hospital information system and reviewed periodically.

Interception Mechanism: Prescriptions cannot be submitted until approved.

Section S3. Interface structure for smart medicine cabinets, PDAs, and the hospital information system

Smart medication cabinet description: The smart medication cabinet used in the intervention was a network-connected controlled-substance cabinet linked to the hospital information system through intermediate SQL Server tables. It supported medication access authorization, drawer/slot-level transaction recording, batch-number linkage, abnormal-return records, local caching during temporary network interruption, and automatic synchronization after network recovery. The smart

medication cabinet used in the intervention was a NuboMed M2300 system manufactured by Shenzhen NuboMed Technology Co., Ltd. The institution-specific configuration was selected because it supported controlled-substance traceability, mandatory verification, offline capture, and auditability within the existing hospital environment. The interfaces were institution-specific integrations using SQL Server intermediate tables and local SQLite caching rather than a publicly documented interoperability standard. Public source code and software-license documentation were not available because the system was institutionally and vendor configured. The hospital retained governance over the clinical workflow, operational records, and locally approved decision-support rules. No intellectual-property transfer or commercial co-development arrangement was part of this study.

Data Interaction Architecture Diagram

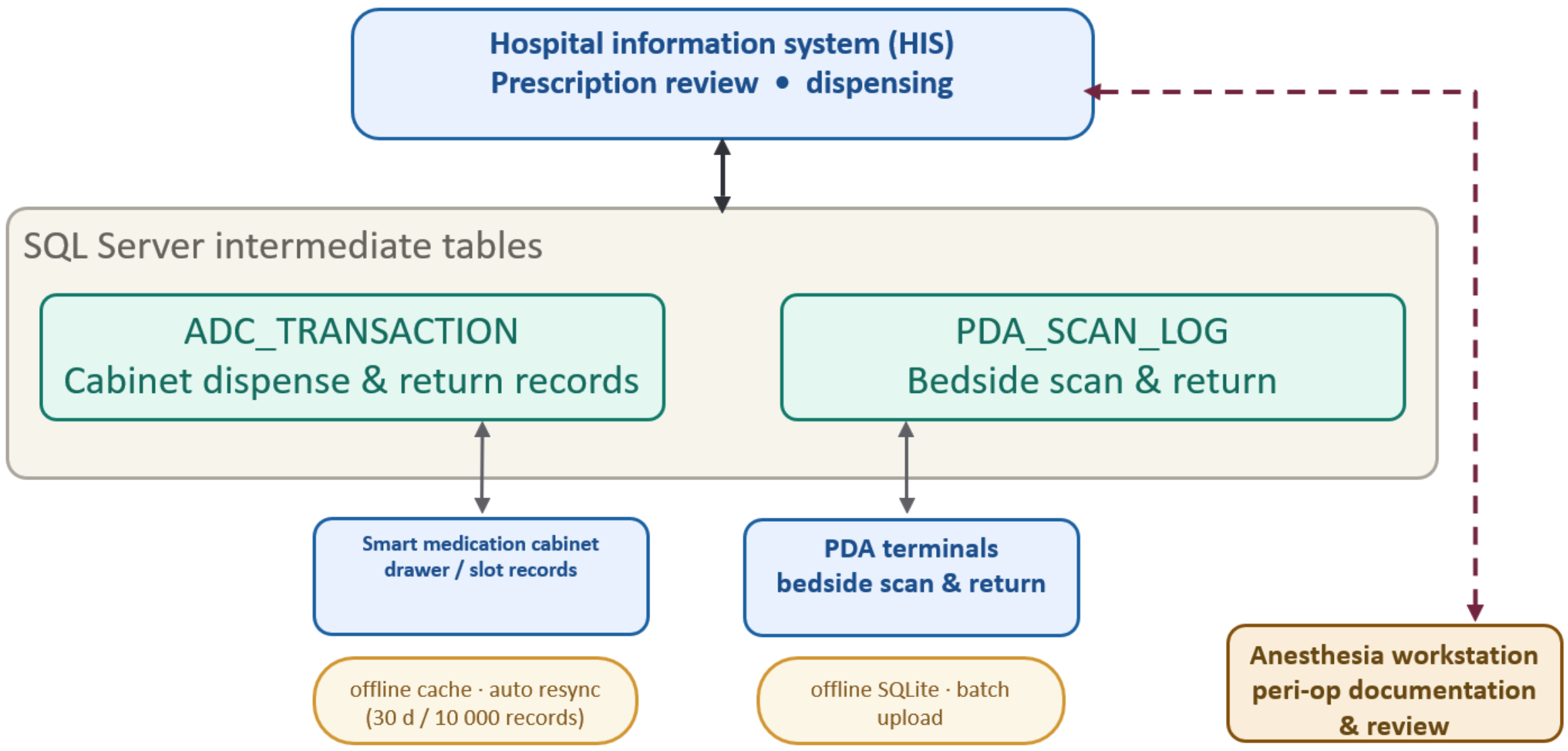

Figure S2. English version of the data-interaction architecture among the hospital information system, intermediate tables, smart medication cabinet, PDA terminals, and anesthesia workstation.

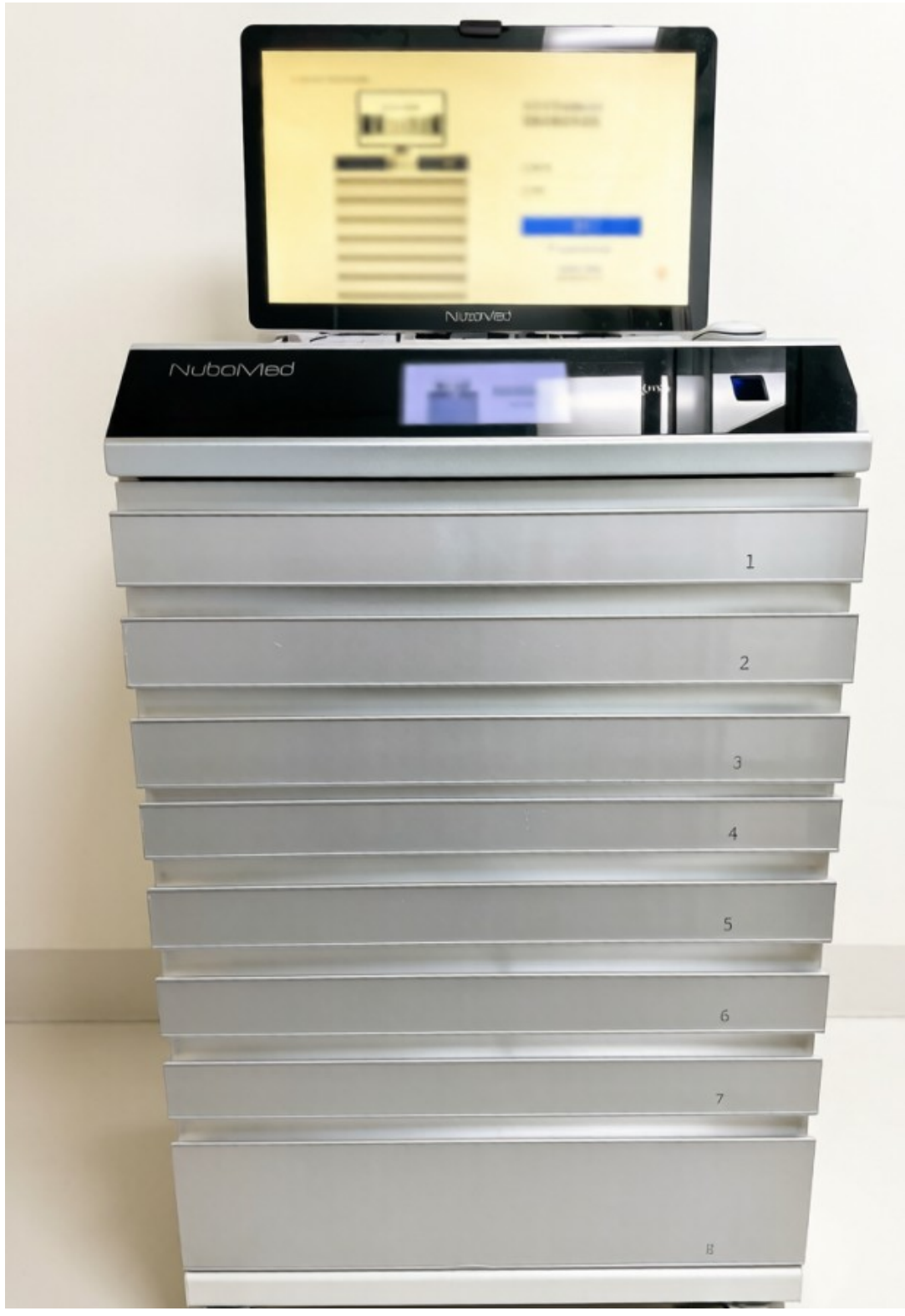

Figure S3. Deidentified exterior photograph of the smart medication cabinet used in the study (NuboMed M2300, Shenzhen NuboMed Technology Co., Ltd.).

Core intermediate table structures

Automated dispensing cabinet (ADC) transaction database table structure

Field Name	Type	Description
TransactionID	varchar(50)	Unique transaction ID, primary key
CabinetCode	varchar(20)	Medicine Cabinet ID
DrawerSlot	varchar(10)	Drawer Slot Code
DrugCode	varchar(30)	In-house drug code
LotNumber	varchar(30)	Lot Number
Quantity	int	Quantity Dispensed
OperatorID	varchar(20)	Operator ID
PatientID	varchar(20)	Associated patient ID (from medical orders)
OrderID	varchar(50)	Order ID

Field Name	Type	Description
TransactionTime	datetime	Pickup Time
SyncStatus	tinyint	0=Not synchronized, 1=Synchronized

PDA_SCAN_LOG database table structure

Field Name	Type	Description
ScanID	varchar(50)	Scan record ID
ScanType	varchar(20)	Type: dispense/admin/return/dispose; dispose status was documented during daily reconciliation or anomaly closure
BarcodeData	varchar(200)	Original barcode content
PatientWristCode	varchar(50)	Patient wristband code (at the time of medication administration)
OrderID	varchar(50)	Associated Order ID
DrugCode	varchar(30)	Drug Code
LotNumber	varchar(30)	Lot Number
ScanResult	varchar(20)	pass/fail
FailReason	varchar(200)	Reason for failure (e.g., batch number mismatch)
OperatorID	varchar(20)	Operator
ScanTime	datetime	Scan Time

Field Name	Type	Description
UploadFlag	bit	0=Not uploaded, 1=Uploaded

Offline Synchronization Logic

PDA Offline Mode: Each scan generates a local SQLite record, which is uploaded in batches to the intermediate table PDA_SCAN_LOG once network connectivity is restored.

Smart Medicine Cabinet Offline Mode: Stores the most recent 30 days of transaction data locally (up to 10,000 records). Once network connectivity is restored, data is synchronized incrementally to ADC_TRANSACTION based on timestamps.

Conflict Resolution: The offline timestamp takes precedence. If a conflicting record (with the same TransactionID) already exists in the HIS during synchronization, the local record is overwritten and marked as "Resend."

Section S4. Tiered alert mechanism in the dispensing and administration process

Expiration-control note: The system distinguished expired medication from medication approaching expiration. Expired medication was treated as a Level 1 mandatory block with no dispensing allowed. Medication with less than 7 days of remaining shelf life triggered a Level 2 conditional hard stop; dispensing could be saved only after confirmation with the receiving ward and two-person authorization, and the override reason was retained in the audit trail.

Alerts are categorized into three levels, ranging from mandatory interception to advisory notifications. These alert levels govern dispensing and administration workflow checks and are separate from the indication-mismatch severity ranking described in Section S2.

Definition of Warning Levels

Level	Name	Trigger Conditions	System Action	User Action
Level 1	Mandatory Block	Batch number mismatch, expired medication, incorrect patient identity, medication does not match the prescription	Screen locks, red pop-up appears, cannot proceed	Must contact a pharmacist or supervisor to unlock, or rescan

Level	Name	Trigger Conditions	System Action	User Action
				the barcode
Level 2	Conditional Block	Empty ampoule with no corresponding usage record, medication marked as damaged, medication not intended for this patient (e.g., picked up by mistake)	Pop-up warning; must check "Known Risk" and enter employee ID to confirm	After confirmation, the process may continue, but the action will be logged
Level 3	Informational Reminder	Non-preferred formulation (e.g., high concentration) or medication stored in an abnormal location	Yellow floating prompt; does not block the operation	Can be ignored; the system only records the number of alerts

Tiered Alerts in the Dispensing Process

The dispensing workflow consists of three steps: scan the prescription barcode, scan the medication unique identifier, and let the HIS perform an automatic comparison.

Validation criteria	Alert Level	Specific Logic
Does the drug batch number match the prescription batch number?	Level 1	If they do not match, the system locks the record, notifies the pharmacist, and requires dual verification to unlock
Is the medication expired or near expiry?	Level 1 for expired medication; Level 2 for remaining shelf life <7 days	Expired medication (expiration date passed) > locked; dispensing prohibited. Medication with remaining shelf life <7 days > conditional hard stop; dispensing can be saved only after ward-supply confirmation and two-person authorization, with the reason logged.
Has the medication been marked as "damaged/returned"?	Level 2	Pop-up: "This medication has been marked as abnormal. Do you wish to proceed?" → A reason was required before proceeding.

Validation criteria	Alert Level	Specific Logic
Does the medication strength match the prescribed dosage?	Level 3	If the prescription states “10 mg” but the medication strength is “50 mg,” a prompt appears: “Possible dosage error”

Tiered Alerts in the Medication Administration Process

The medication-administration workflow consists of four steps: scan the patient wristband, scan the prescription barcode, scan the medication barcode, and complete triple verification.

Verification Items	Alert Level	Specific Logic
Patient identity mismatch	Level 1	Wristband code does not match the patient ID in the medical order → Locked; medication cannot be administered
Medication batch number does not match the dispensing record	Level 1	Prompt: "Batch number does not match. Please retrieve the medication again," blocking administration
Medication has exceeded the prescribed administration window (±30 minutes)	Level 2	Pop-up window: "Administration time exceeded; please confirm patient status." Requires confirmation by checking a box
The same patient has already received a similar medication (e.g., fentanyl + sufentanil)	Level 2	Pop-up: "Possible duplicate medication," requires physician confirmation
Remaining shelf life of medication < 7 days	Level 2	Conditional hard stop: the system displays a near-expiry warning and blocks routine release. The operation can continue only after confirmation with the receiving ward and two-person authorization;

Verification Items	Alert Level	Specific Logic
		the authorization reason and operator IDs are retained in the audit log.

Section S5. Daily reconciliation checklist and standard operating procedure for anomaly handling

Daily Settlement Reconciliation Checklist

No.	Check Item	Data Source	Verification Method	Handling of Discrepancies
1	Total Prescriptions for the Day	HIS prescription records	Compare with pre-authorization interception records	Intercepted prescriptions were logged; discrepancy ≤ 0.1%
2	Total number of dispensed medications for the day (units)	Smart Medicine Cabinet Dispensing Records	Compare with PDA dispensing scan records	Trace back to the specific operator in case of discrepancies
3	Total number of medications administered on the day (units)	PDA Medication Administration Scan Records	Compare with dispensing records (at the lot number level)	Batch number mismatches were corrected within 24 hours
4	Total number of empty ampoules returned on the day (units)	PDA Return Scan Records	Compare with administration records (batch-level)	List of unreturned vials was sent to the head nurse and entered within a specified timeframe
5	Batch Number Discrepancy Report	Automatically generated by the system	Export Batch Status Closure Report	Batch numbers with the status "Dispensed but Not Returned" require a written explanation
6	Damaged/Returned Medication Records	Abnormal Incident Handling Module	Dual-Signature Electronic Archiving	Monthly Summary and Analysis
7	Smart Medicine Cabinet	Real-time cabinet	Comparison with HIS	Initiate a reconciliation

No.	Check Item	Data Source	Verification Method	Handling of Discrepancies
	Inventory Levels	data	Book Inventory	when the discrepancy is ≥1%

Verifiers: On-duty Pharmacist + Reviewing Pharmacist

Deadline: The system automatically generates reports at 24:00 daily; reconciliation and electronic signature are due by 10:00 the following day.

Standard Operating Procedure for Anomaly Handling

Medication Return

Scenario: An unopened medication that has been dispensed but not used is returned to the pharmacy.

Procedure:

The nurse selects the “Medication Return” function on the PDA and scans the medication barcode;

The system checks whether the medication has been opened (based on the packaging integrity status; the nurse must check “Packaging Intact”);

If unopened, the system generates a medication return form and prints two copies;

Both the nurse and pharmacist sign the form; one copy is retained at the nurses’ station, and the other is submitted to the pharmacy;

The pharmacist scans the barcode on the return form and the medication barcode, and the system automatically updates the inventory in the smart medicine cabinet;

A return log is recorded (operator, time, reason, lot number).

Opened medications were not returned and were treated as damaged.

Damage

Scenario: Vial breakage, label damage, or a similar issue occurs during storage or transportation.

Procedure:

The person who discovers the issue must immediately take a photo and upload it to the HIS Incident Reporting module;

Two personnel (pharmacist + nurse or pharmacist + pharmacist) must verify the issue on-site;

Select “Damage Registration” on the PDA, scan the medication barcode (if scannable) or manually enter the traceability code;

The system marks the medication as “Damaged—Disposed,” deducts it from inventory, and generates a damage record;

Place the damaged medication in a dedicated “Damaged Medication Collection Box” for centralized monthly disposal (supervised by two personnel);

Compile the monthly damage rate; analyze the cause if it exceeds the threshold (>0.05%).

Emergency Medication Borrowing Outside Business Hours

Scenario: A controlled-substance medication is borrowed from the emergency window outside business hours.

Procedure:

A physician and a nurse on duty proceed to the pharmacy emergency window together;

Both undergo dual authentication via fingerprint and password (pharmacy emergency system);

The system automatically generates an “Emergency Medication Dispensation Record,” which requires entering the patient ID, medication name, quantity, and lot number;

After dispensing the medication, a pharmacist must retroactively enter the prescription and dispensing records into the HIS within 24 hours;

If not entered within 24 hours, the system automatically sends an alert to the Director of Pharmacy and locks the physician’s medication borrowing privileges.

Authorization is limited to on-duty staff in Anesthesiology, Emergency Medicine, and the intensive care unit (ICU), and the list is updated monthly.

Table S1. Contextual workload and staffing indicators during the pre- and post-intervention periods

This table provides descriptive context for workload, staffing, and department mix during the two observation windows. The workload indicator is the number of surgical patients based on unique admission numbers. These indicators do not establish equivalence between periods or eliminate residual temporal confounding from seasonality, the New Year holiday, or the 2025 Spring Festival

period. Staffing indicators represent the actual numbers of staff members assigned during each study period, not cumulative duty shifts.

Metric	Unit	Pre-intervention period (Sep 1-Nov 30, 2024)	Post-intervention period (Dec 1, 2024-Feb 28, 2025)	Absolute difference	Data source	Notes
Surgical patients based on unique admission numbers	No.	4837	4191	-646	Source-verified surgical workload dataset derived from HIS and anesthesia information system	These values describe workload context only and do not establish equivalence between periods or eliminate residual temporal confounding.
Inpatient bed occupancy	%	85.5	86.7	+1.2 percentage points	Hospital administrative monthly reports	The hospital administrative reporting definition was used; this indicator was not recalculated from inpatient-days and open beds.
Open beds	No.	1150	1150	0	Medical affairs department	The number of open beds was unchanged across the two periods.
Active prescribing departments	No.	34	34	0	Medical affairs department	The range of departments prescribing controlled substances was unchanged.
Prescription share of the top 5 prescribing departments	%	61.2	60.8	-0.4 percentage points	Prescription-review system	The top 5 departments accounted for a similar proportion of controlled-substance prescriptions, suggesting a stable department mix.
Controlled-substance prescription volume	No.	3264	3311	+47	Prescription-review system	The denominator includes the operating-room anesthesia pharmacy and is consistent with Table 3 in the main manuscript.
Assigned pharmacists	No. of	22	22	0	Pharmacy	Period-specific assigned headcount; not cumulative

(headcount)	staff members				department roster	duty shifts or FTEs.
Assigned physicians (headcount)	No. of staff members	204	203	-1	Medical affairs department	Period-specific assigned headcount; not cumulative duty shifts or FTEs.
Assigned nurses (headcount)	No. of staff members	521	512	-9	Nursing department roster	Period-specific assigned headcount; not cumulative duty shifts or FTEs.

Abbreviations: HIS, hospital information system.

Table S2. Data-quality verification and sensitivity analyses

This table reports the source-verified counts used in the manuscript. Four operational records were flagged during source verification: two pre-period records were source-confirmed and retained in the primary analysis, one post-period duplicate record was excluded to avoid double counting, and one post-period incomplete record was excluded because required fields were missing. The primary analysis used the resulting source-verified dataset.

Outcome	Primary pre n/N (%)	Primary post n/N (%)	Primary absolute difference, percentage points	Primary P value	Sensitivity pre n/N (%)	Sensitivity post n/N (%)	Sensitivity absolute difference, percentage points	Sensitivity P value	Records affecting the outcome
Prescription-dispensing record compliance	3095/3264 (94.82%)	3268/3311 (98.70%)	+3.88 (95% CI 3.03 to 4.73)	P < .001	3093/3262 (94.82%)	3268/3311 (98.70%)	+3.88 (95% CI 3.03 to 4.73)	P < .001	Leave-two-records-out sensitivity analysis excluded these two records.
Batch-number management non-compliance	24/1000 (2.40	6/1000 (0.6	-1.80 (95% CI -2.86 to	P = .001	24/1000 (2.40%)	6/1000 (0.60	-1.80 (95% CI -2.86 to	P = .001	Sample-based outcome; source review did not

	%)	0%)	-0.74)			%)	-0.74)		change the total.
Failure to complete empty-ampoule recovery and electronic documentation within 24 hours	68/1213 (5.61%)	10/1274 (0.78%)	-4.82 (95% CI -6.20 to -3.44)	P < .001	68/1213 (5.61%)	10/1274 (0.78%)	-4.82 (95% CI -6.20 to -3.44)	P < .001	One post-period record was excluded because it was a duplicate and could have caused double counting; one post-period record was excluded because required fields were incomplete.

Four operational records were flagged during source verification. Two pre-period records were source-confirmed and retained in the primary analysis, one post-period duplicate record was excluded to avoid double counting, and one post-period incomplete record was excluded because required fields were missing. The leave-two-records-out sensitivity analysis excluded the two source-confirmed pre-period records, reducing the compliance denominator from 3264 to 3262. Between-period absolute-difference 95% confidence intervals were calculated using the Wald normal approximation for independent proportions.

Table S3. Exploratory drug-type subgroup analyses at the prescription-dispensing record level

This table reports prespecified drug-type subgroups with reliable denominators at the prescription-dispensing-record level. Drug-type subgroup definitions followed the medication categories used in the hospital prescription-review system. Department-type subgroup estimates were not retained because a matched deidentified denominator was not available.

Outcome	Subgroup	Pre n/N (%)	Post n/N (%)	Absolute difference, percentage points (95% CI)	P value	Interaction P value
Compliance of prescription-dispensing records	Fentanyl-related drugs	82/85 (96.47%)	184/191 (96.34%)	-0.14 (-4.88 to 4.61)	P = .955	Not performed; descriptive only.
Compliance	Sufentanil	493/528	573/579	+5.59 (3.32 to	P <	Not performed;

of prescription-dispensing records		(93.37%)	(98.96%)	7.87)	.001	descriptive only.

Note: Subgroup analyses were exploratory record-level analyses, and no formal interaction test was performed. The subgroup-specific estimates should therefore be interpreted descriptively and not as evidence of effect modification. Counts were calculated using the same prescription-dispensing-record analytic unit as the primary outcome analysis. Drug-type classification followed the medication categories used in the hospital prescription-review system.

Table S4. Inter-rater reliability based on independent dual-reviewer ratings

Cohen's kappa was calculated from a prespecified stratified random sample of 500 source-verified analytic records in each outcome domain (250 pre-intervention and 250 post-intervention records per domain). The calculation used only the two independent reviewer-rating columns before adjudication; final adjudication/status results were not used to compute kappa. The two reviewers were independent and blinded to each other's ratings.

Outcome domain	Valid A/B pairs, n	Agreement, n	Agreement rate	Cohen's kappa	A/B disagreements, n
Prescription-dispensing record compliance	500	498	99.6%	0.898	2
Batch-number management	500	498	99.6%	0.956	2
Empty-ampoule recovery	500	499	99.8%	0.964	1
